# Multiple Frequencies Sequential Coding for SSVEP-Based Brain-Computer Interface

**DOI:** 10.1371/journal.pone.0029519

**Published:** 2012-03-06

**Authors:** Yangsong Zhang, Peng Xu, Tiejun Liu, Jun Hu, Rui Zhang, Dezhong Yao

**Affiliations:** Key Laboratory for NeuroInformation of Ministry of Education, School of Life Science and Technology, University of Electronic Science and Technology of China, Chengdu, China; Cuban Neuroscience Center, Cuba

## Abstract

**Background:**

Steady-state visual evoked potential (SSVEP)-based brain-computer interface (BCI) has become one of the most promising modalities for a practical noninvasive BCI system. Owing to both the limitation of refresh rate of liquid crystal display (LCD) or cathode ray tube (CRT) monitor, and the specific physiological response property that only a very small number of stimuli at certain frequencies could evoke strong SSVEPs, the available frequencies for SSVEP stimuli are limited. Therefore, it may not be enough to code multiple targets with the traditional frequencies coding protocols, which poses a big challenge for the design of a practical SSVEP-based BCI. This study aimed to provide an innovative coding method to tackle this problem.

**Methodology/Principal Findings:**

In this study, we present a novel protocol termed multiple frequencies sequential coding (MFSC) for SSVEP-based BCI. In MFSC, multiple frequencies are sequentially used in each cycle to code the targets. To fulfill the sequential coding, each cycle is divided into several coding epochs, and during each epoch, certain frequency is used. Obviously, different frequencies or the same frequency can be presented in the coding epochs, and the different epoch sequence corresponds to the different targets. To show the feasibility of MFSC, we used two frequencies to realize four targets and carried on an offline experiment. The current study shows that: 1) MFSC is feasible and efficient; 2) the performance of SSVEP-based BCI based on MFSC can be comparable to some existed systems.

**Conclusions/Significance:**

The proposed protocol could potentially implement much more targets with the limited available frequencies compared with the traditional frequencies coding protocol. The efficiency of the new protocol was confirmed by real data experiment. We propose that the SSVEP-based BCI under MFSC might be a promising choice in the future.

## Introduction

Steady-state visual evoked potential (SSVEP) is a continuous sequence of oscillatory potential changes elicited in the visual cortex when a repetitive or flickering visual stimulus is presented to a subject [1]. The SSVEP is usually a near-sinusoidal waveform with the same fundamental frequency of the driving stimulus as well as its harmonics, and generally appears in occipital and parietal lobes.

In recent years, due to the short response time, the high information transfer rate (ITR), the noninvasiveness, and the inherent response of the brain, SSVEP has been used as the typical input signal for BCI system [2], [3], [4], [5], [6].Like BCI systems based on motor imagery [7], [8] and P300 [9], [10], SSVEP-based BCI has become a crucial branch of BCIs.

In the SSVEP-based BCI, visual stimulators elicited SSVEP is the fundamental working mechanism of the system. Several visual stimulators have been used for evoking SSVEP, such as a cathode ray tube (CRT) monitor, liquid crystal display (LCD) monitor, and light-emitting diode (LED) array [5]. The flickering stimuli of different frequencies with a constant intensity will evoke the SSVEP having different amplitude strengths. Usually, the strongest SSVEP, the medium strong SSVEP and the weak SSVEP could be observed by those stimuli in the low frequency range 5–12 Hz, in the medium range 12–25 Hz and in the high frequency range 25–50 Hz, respectively [11]. If the visual stimuli is displayed on the LCD or CRT monitors, the stimulus frequencies are limited by the refresh rate of the stimulators, and the lack of available modulation frequencies is a problem to be solved in SSVEP-based BCIs [12], [13], [14], [15], [16]. Even if the LED stimulators are able to evoke an SSVEP in a frequency range of 1–90 Hz [11], for a subject, the available frequencies that can evoke the detectable SSVEP are still limited due to the still unclear physiological mechanism. Spelling was one of the first and may be the most basic BCI application, which remains a benchmark for communication application and one major challenge in the BCI community [15], [17]. For SSVEP-BCIs, the stimuli with traditional single frequency coding protocol were not enough to be assigned to every character for the speller interface on LCD or CRT stimulators [15]. Therefore, it may be a critical challenge for the design of a practical SSVEP-based BCI to code more targets under limited frequencies.

To tackle the problem, some groups have used the phase information to code the targets in the SSVEP-BCI. The related studies show that this kind of coding protocol can actually increase the coding targets with limited available frequencies [14], [16]. For example, Jia et al [16] realized a BCI system with 15 targets with three stimulus frequencies using the frequency and phase mixed information to increase possible target number.

Other two groups used the different protocols to increase the number of flickering targets [12], [13]. In these two systems, each target was simultaneously modulated by two different frequencies, which can generate more flickering targets due to the combination of frequencies. Because only the combination of frequencies is used in this kind of coding protocol, the coding target number cannot be compared to that when the information of flickering order is adopted.

To code more targets using multiple frequencies, in this paper, we present a novel multiple frequencies sequential coding (MFSC) protocol for SSVEP-based BCIs. Different from the simultaneous multi-frequency flickering protocol, MFSC uses sequential multiple frequencies flickering to code the targets. The efficiency of the new protocol was confirmed by real experimental dataset. The paper is organized as follows. Results are provided in Section *Results*. Section *Discussion* is a general discussion for the results. Section *Materials and Methods* provides a detailed description of MFSC, stimulator and experiment setups, target recognition for MFSC and evaluation index, are included in this section too.

## Results

All experimental data were re-sampled to 250 Hz and filtered with a band-pass of 4–40 Hz for off-line analysis. Each condition contained eighty trials for each subject, and accordingly each target of each condition had 20 trials. The canonical correlation analysis (CCA) is used for frequency detection with signals from channels of P3, P4, O1 and O2 as its input. O1 and O2 were in the occipital region, and the reason to involve P3, P4 was because the parietal region is the important information hub for SSVEP [18], where obvious SSVEPs can also be observed. In reference [19], the authors discussed the relationship between the recognition accuracy and the number of harmonics included in the reference signals, and they found that two harmonics in their online experiment are adequate to achieve a reliable result. Based on this, we also used two harmonics to construct the reference signals in our off-line data analysis.

The discrimination accuracies of the ten subjects for the two different conditions and the corresponding average accuracy for the two conditions were shown in Table 1.

**Table 1:** Classification accuracies(%) of the subjects under the two different conditions.

Subjects	Accuracy	
	2 s	1.5 s
**S1**	98.75	95.00
**S2**	98.75	97.50
**S3**	100.00	98.75
**S4**	92.50	82.50
**S5**	98.75	95.00
**S6**	88.75	82.50
**S7**	93.75	92.50
**S8**	92.50	78.75
**S9**	95.00	86.25
**S10**	100.00	92.50
**Average(std)**	95.87(3.91)	90.13(7.06)

As shown in Table 1, the accuracies of 2 seconds condition were above 90% for nine subjects. Accuracies of 1.5 seconds condition were above 80% for nine subjects with six of them above 90%. For all subjects, the accuracy was improved when the longer duration time 2 seconds was used, especially for S4, S8, S9, S10.

The ITR was used as a standard measure to evaluate the performance of BCI systems [20]. For our off-line analysis, we used the same simulation method proposed in [16] to conduct a simulated online test. For the two different conditions, we set the *t*_3_ as 0.5 s [16] as the rest time given to the subjects to shift gaze to simulate the online situation. Table 2 listed the simulated ITR calculated for the two different conditions for each subject.

**Table 2:** Simulated on-line ITR (bits/minute).

Subject	ITR	
	2 s	1.5 s
**S1**	25.11	28.02
**S2**	25.11	30.72
**S3**	26.67	32.28
**S4**	19.96	18.06
**S5**	25.11	28.02
**S6**	17.52	18.06
**S7**	20.85	25.66
**S8**	19.96	15.72
**S9**	21.79	20.65
**S10**	26.67	25.66
**Mean(std)**	22.87(3.24)	24.28(5.78)

Considering the more complex situation in practical application, the ITR in an on-line system may differ from our simulated results.

## Discussion

### 1 MFSC

The limited number of available frequencies due to both the hardware refresh rate and the human neurophysiological mechanism precludes the development and application of SSVEP-based BCIs. For traditional single frequency coding protocol, one target needs one frequency, and therefore the frequency for different target must be different [4], [5]. For the simultaneous dual frequencies coding protocol which has been proposed for the LED stimulators [13], the combination of frequencies are used for coding, where the frequencies forming combination must be different and different targets need different frequency combinations [12], [13]. To our best knowledge, if this protocol is used [12], [15], the number of targets still cannot satisfy the requirements of actual BCI application. By adding the temporal factor into coding protocol, we developed the MFSC protocol that used the permutation of frequencies to code more targets with the limited available frequencies. Under MFSC protocol, each target was coded by one permutation sequence, where the frequencies coding this target are presented one by one according to the order in the permutation sequence. Accordingly, MFSC allowed the utilization of the same frequencies combination to code different targets, which cannot be realized by the simultaneous dual combination. If the length of coding sequence is kept to 2, the target number is the square of the number of the available frequencies. With six frequencies, thirty-six targets can be generated, which is comparable to the common P300 based speller system [9]. Moreover, the speller system realized with MFSC protocol can code every character directly unlike the indirectly coded system in [15], [17], where a strategy has to be found for combining the basic commands due to the lack of enough targets, and the time needed for a selection will be longer than the directly coded system [15].

In Table 3, we listed the target number that can be coded when different number of frequencies (*N*) are used under different coding protocols. It is clear that MFSC with coding length 2 (*M* = 2) can code most targets among the three protocols.

**Table 3:** The target number that can be coded when different number of frequencies are used under different coding protocols.

Coding Method	Frequencies Number						
	2	3	4	5	6	…	*N*
**SF**	2	3	4	5	6	…	*N*
**DFC**	3	6	10	15	21	…	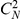 +N
**MFSC**	4	9	16	25	36	…	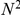

SF represents the traditional single frequency coding, and DFC shows the dual frequencies combination coding. The coding length for MFSC is 2.

By increasing the coding length of MFSC, more targets can be coded. But this will increase the coding time in one flickering cycle, and ultimately increases the detection time for the targets, reducing the ITR of the proposed system. As revealed by the above Table 3, the enough targets for practical BCI application can be achieved only with 2-length MFSC.

For the traditional dual frequencies simultaneous coding method, to acquire the reliable dual SSVEP components from EEG signals, the dual frequency of the stimuli needs to be at least 4 Hz apart [21], which may further lower the number of available frequencies to code the targets. But this limitation is not a problem for MFSC, because the multiple frequencies of MSFC are relatively independently presented in the sequential form.

In previous studies [16], [19], the authors had proposed a short period of time(0.5 s [16], 0.3 s [19]) to allow the users to shift their gaze after a selection. Similarly, in MFSC, we introduced the break time between each cycle, which may result in less annoyance to the subjects. Of course, the longer the break time is, the lower ITR the system will have. In practical application, under the acceptable speed of the system, the break time can be determined according to the specific requirement of the users.

### 2 Frequency recognition Method

Recently, multiple channel EEG signals were used in SSVEP-based BCI for frequency recognition [17], [19], [22], [23]. With multiple channel classification methods, no calibration is needed for electrode selection for each subject, which brings some convenience for the application compared to other methods [24], [25]. In our off-line analysis, four electrodes (P3, P4, O1 and O2) that are of strong SSVEPs were chosen for frequency recognition.

In this study, we just used the CCA method for SSVEP detection. The two main parameters for the reference signals in the CCA was not optimized for each subject, and optimized parameters and electrode selection may further improve the performance for some subjects, such as subject 6, 8 and 9.

For frequency recognition, although there are some other variations such as the sparse CCA [26], kernal CCA [27], the combination of CCA plus Fast Fourier Transform (FFT) [28], and linear discriminant analysis (LDA) [29] etc., we used the original version of CCA. Our choice is based on the following three reasons: 1) as for BCI practical application, the efficiency and stability are the two most important issues, and the ordinary CCA version has been confirmed to be a very efficient algorithm for both off-line and on-line SSVEP-BCI systems [19], [23], [30], [31], 2) as a primary report of MFSC, the main purpose of this paper is to show the coding principle;3) the ordinary version of CCA is easy to implement for all the three approaches for a fair comparison. However, to best show the potential of MFSC in the future, we will consider some other recently reported frequency recognition methods.

### 3 The interaction between two sequential frequencies

In most SSVEP based BCI system, each target is coded just by one frequency. But with MFSC of 2-length coding, two frequencies were involved to code target by their sequential flickering, and one question may be raised that whether the first frequency in *t_1_* epoch may influence the recognition of the second frequency in *t_2_* epoch, in the other words, if the detection of the second frequency will be of an higher error rate. In order to check this possible interaction between the two sequential frequencies, we will separately perform the frequency recognition with CCA for epochs *t_1_* and *t_2_*, and the accuracies for frequency detection in these two epochs are listed below in Table 4.

**Table 4:** Frequency recognition accuracies (%) for epochs *t_1_* and *t_2_* in the 2-length MFSC.

Subject	Epoch(Time condition)			
	*t_1_* (2 s)	*t_2_* (2 s)	*t_1_* (1.5 s)	*t_2_* (1.5 s)
**S1**	100.00	97.50	97.50	95.00
**S2**	100.00	97.50	100.00	95.00
**S3**	100.00	100.00	100.00	100.00
**S4**	95.00	95.00	95.00	100.00
**S5**	97.50	97.50	95.00	100.00
**S6**	95.00	92.50	85.00	95.00
**S7**	95.00	100.00	97.50	97.50
**S8**	97.50	97.50	82.50	100.00
**S9**	97.50	97.50	92.50	87.50
**S10**	100.00	100.00	92.50	95.00
**Average(std)**	97.75(2.19)	97.50(2.36)	93.75(5.92)	96.50(3.94)

We used the paired *t*-test to investigate if the accuracy difference between *t_1_* and *t_2_* epochs are of statistical sense. The statistical test reveals that no significant difference exists between the two epochs, where *p*>.70 for 2 s-long epoch and *p*>.20 for 1.5 s-long epoch. Based on this, we can roughly conclude that the prior frequency in epoch *t*_1_ has no significant effect on the recognition of the following frequency in epoch *t*_2_.

### 4 The effect of data length and used frequencies on performance

In real time system, there is a tradeoff between the accuracy and speed which is relative to the data window length. The more data is used in the CCA, the higher accuracy can be attained [19], [23], which is revealed in Table 1. Though the longer time window facilitate to the frequency recognition, the longer time will be needed to perform one selection, which may decrease the system ITR. As revealed in Table 1, when the stimulus cycle is 3 s (1.5 s long epoch), the average accuracy for the ten subjects can be comparable to the results reported in [24], [25]. As for a practical BCI system, a 3–4 s long recognition time is acceptable [17]. The results in Table 1 confirmed that a longer stimulus duration may facilitate the classification, where the 2 s duration for each frequency has a better performance than the 1.5 s situation. However, in the current work, the durations in one stimulus cycle for different frequencies are kept to be equal (*t*_1_ = *t*_2_), and we will optimize the coding time for different frequencies in the future, which may further reduce the recognition time for one target.

The frequencies used to code target may influence the recognition performance to some degree because different subjects may have their favored frequencies [4], [23], which should be optimized for each subject. In current work, we did not perform the frequency optimization for each subject and only simply use 7.5 Hz and 12 Hz for all subjects. As shown in Table 1, the accuracy for the subject 6 was relatively lower compared to the other subjects, which may be due to the fact that the used frequencies cannot evoke the strongest SSVEPs for this subject. If the favored frequencies can be selected before the experiment, the performance of MFSC could be further improved.

### 5 The effect of user's familiarity on target recognition

**Figure 1:**
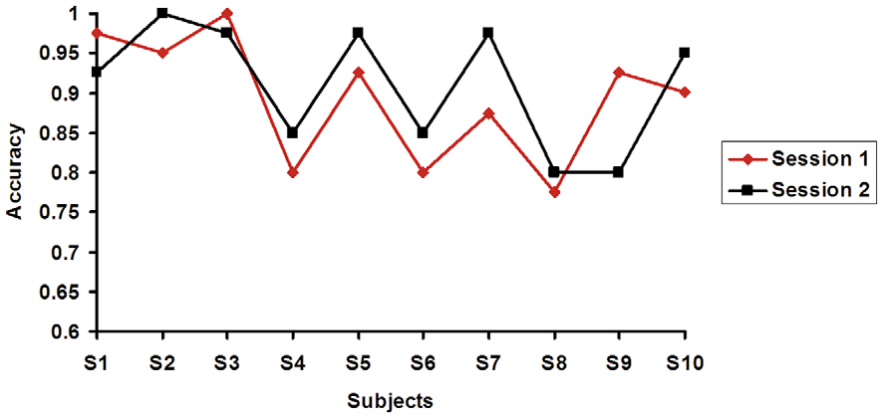
The accuracies of *t*_1_ = *t*_2_ = 1.5 s in the separate session.

**Figure 2:**
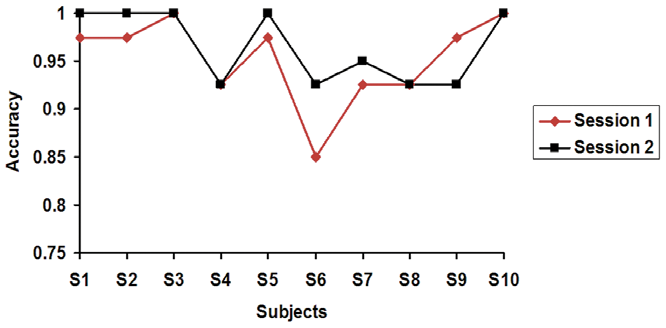
The accuracies of *t*_1_ = *t*_2_ = 2 s in the separate session.

The experiment consists of two sessions, and the overall accuracy of the two sessions under different epoch durations are listed in Table 1. To check if there exists performance difference between the two sessions, the recognition accuracies in the two sessions for the two different epoch durations are shown in Figure 1 and Figure 2, respectively. The performance in the two sessions of the subjects was not consistent. For most subjects, the performance in session 2 was better than that in the session 1, this may attribute to the improved attention strategy, because attention can modulate the SSVEP [32]. Except for subjects 1, 3 and 6, all other subjects are native to SSVEP-based BCI paradigm, and we think the experience in session 1 can provide training for those subjects to control the SSVEP system. For some of the subjects, the performance in session 1 was better than that in session 2, and we think this may attribute to the subject' fatigue.

### 6 Information Transfer Rate

The ITR of SSVEP-BCI mainly depends on three factors, i.e., the total number of targets in the system, the accuracy and the time needed to produce a selection. The ITR of the simulated on-line tests for the two conditions are 22.87(3.24) and 24.28 (5.78) bits/minute, which is comparable to some of the existed systems with ITR 27.15bits/min (This system had thirteen targets.) [4] and 28.29±12.19 bits/min (This system had eight targets) [14]. As mentioned above, with six frequencies, the MFSC of length-2 can realize 36 targets at the 2-length code, the ITR corresponding to the 36 targets system would be improved much. This needs to be validated in the future research.

### 7 Asynchronization of SSVEP-based BCI under MFSC

As mentioned above, the stimulator sends three tags in each stimulus cycle to the data acquisition system, which can guarantee the two systems are synchronized. The online classification algorithm only needs to process the data in epochs *t*_1_ and *t*_2_ in each stimulus cycle, which can be finished in the break epoch *t*_3_. When some commands need to be sent with the BCI under MFSC protocol, the user just needs to fixate on the targets. Obviously, when the subject begins to fixate on the target just at the onset of epoch *t*_1_, the target recognition can be finished in one cycle. However, if the time that the user begins to fixate on target is not consistent with onset of epoch *t*_1_, the command may be sent out with a cycle lag, which may be different from the traditional asynchronous BCI system. In the MSFC coding based system, the lag time is dependent on the cycle duration and just several seconds, which still is acceptable for a practical BCI system. In the future, with the optimization for both the subject specified frequency and epoch time, the cycle time can be further reduced and the lag time can also be reduced. We can also introduce the brain switch to open and close the stimuli in our future on-line system to improve the asynchronization for the BCI under our proposed protocol [7], [33].

## Materials and Methods

### 1 Ethics statement

This study was approved by the Institution Research Ethics Board at University of Electronic Science & Technology of China. All participants were asked to read and sign an informed consent form before participating in the study. After experiment, all the participants received a monetary compensation for their time and effort.

### 2 MFSC

The limited number of targets under the traditional frequencies coding methods block the application of SSVEP-based BCI systems, and it is urgent to resolve this challenge in this field. In traditional coding methods, the targets are usually coded by the simultaneously presented frequencies, which did not use the temporal information as a parameter to code the targets. Adding the time factor, the traditional frequency-spatial coding protocols will be extended to frequency-temporal-spatial coding strategy. Motivated by this idea, we developed a novel method termed as MFSC with an aim to achieve the more effective coding of targets for SSVEP-based BCI systems.

Under MFSC, each target is coded by the permutation of several frequencies from an available frequencies set. The reported protocols in [12], [13] usually code the targets by the combination of frequencies. In MFSC, those multi-frequencies used to code one target will be sequentially presented. To realize MFSC, a stimulus MFSC coding cycle is introduced, which is defined as one period that the used frequencies can code one target in sequence. Each target is coded by the predefined frequencies cycle by cycle. In each cycle, there are several continuous coding epochs, following a break time epoch. The duration of each epoch can be the same or different from each other. The break time epoch is used as the shift gaze time, and its duration can be set according to the specific requirement of the user. As for the coding procedure for one target, each coding epoch in one cycle will be occupied by certain predefined frequency of an available frequencies set, then the different placement of frequencies in epochs will accomplish different coding for multi-targets. The epochs behave like a time coding chain.

**Figure 3:**
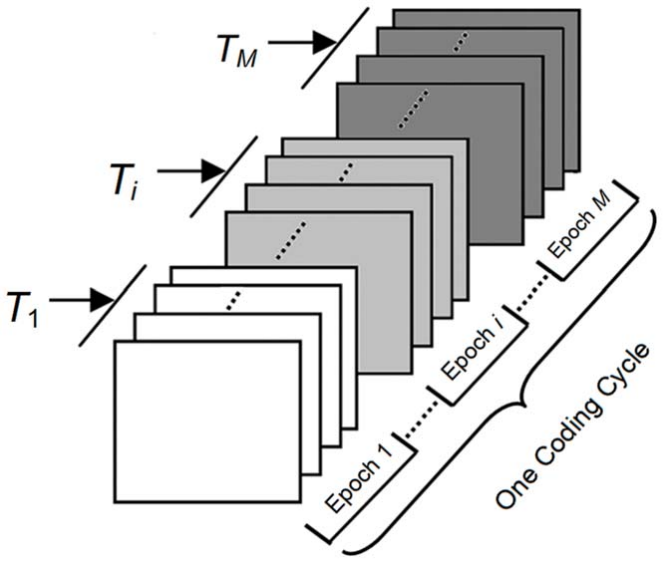
The coding principle of MFSC with *N* frequencies and *M*-length sequence to code one target in one cycle. In this figure, we used the different colors to denote the frequencies in the different coding positions of the *M*-length coding sequence. When the length of the coding sequence is *M*, the used frequency *T*_i_(*i* = 1,..,*M*) can be anyone of the adopted frequencies set (*F* = {*f*_1_, *f_i_*,…, *f_N_* }).

Therefore, in MFSC protocol, the multiple frequencies used to code one target are not simultaneously presented, which is different from the coding methods introduced in [12], [13]. With this scheme, MSFC can code more targets compared to the reported coding protocols. The coding principle of MFSC can be depicted as Figure 3.

In Figure 3, to code one target, there are *M* (*M*>1) coding epochs (denoted with different gray scales) in one cycle, i.e., the length of the coding sequence is *M*, and the break time epoch was not included in this figure. The available frequencies set (*F* = {*f*_1_, *f_i_*,…, *f_N_* }) consisted of *N* frequencies. When the target is coded, *T*_1_, *T_i_*,…, *T_M_* (*T*_i_∈*F,i* = 1,..,*M*)are the frequencies independently selected from the frequencies set(*T*_i_∈*F*), and these frequencies can be totally the same, or partly different, or completely different. Accordingly, in one cycle of a target coding, each epoch will be assigned a frequency selected from the available frequencies set, and only these frequencies corresponding to the M epochs will flicker independently and subsequently.

With the above MFSC protocol, the targets coding are actually realized in a sense of permutation. According to the permutation theory, putting *N* frequencies into *M* positions, we can get *N^M^* permutation sequences. Therefore, if *N* frequencies are used to code targets with *M*-length coding sequence, *N^M^* targets could be coded, which is much larger than the targets that can be achieved by the simultaneous combinations coding method (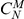
+*N* targets) or the single frequency coding method (*N* targets),as shown in Table 3.

In essence, it is the utilization of time factor that let MFSC to realize the more targets in sense of permutation. With the time factor involved in coding scheme, the traditional frequencies combination SSVEP protocol is extended to a frequency-temporal-spatial coding approach. For example, with 2-length(*M* = 2) coding, two frequencies (*N* = 2) can accomplish the coding of four targets and nine targets can be achieved with three frequencies, whereas the corresponding targets can be realized by the traditional frequencies combination approach are only 3, and 6, respectively. Apparently MFSC does enables us to get more targets than the simultaneous combination method.

**Figure 4:**
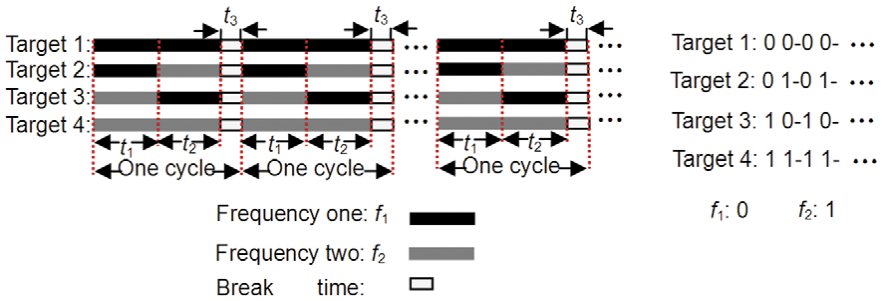
The 2-frequencies sequential coding paradigm. In each coding cycle, the black bar denotes the coding flickering epoch (*t*_1_) for the first frequency (*f*_1_), the gray bar denotes the coding flickering epoch (*t*_2_) for the second frequency (*f*_2_), and the white rectangle denotes the break time epoch (*t*_3_).

Apparently, the longer the code length (*M*) is, the more time is needed for one stimulus cycle, but the targets that can be coded will increase dramatically (*N^M^*). In this study, as an example to code four targets to preliminarily reveal the performance of MSFC, two frequencies (*f*_1_, *f*_2_ : *N* = 2) and two coding epoch(*t*_1_, *t*_2_: *M* = 2) were adopted in each coding cycle. Figure 4 shows the paradigm, where the break time epoch (*t*_3_) is added to each coding cycle.

In Figure 4, we can see that the targets are coded by the two frequencies cycle by cycle in details, target 1 is modulated only by *f*_1_ denoted by two black bars in each cycle; Target 2 is modulated by *f*_1_ and *f*_2_, with *f*_1_ in the first epoch and *f*_2_ in the second epoch; Similar to target 2, target 3 is coded by presenting *f*_2_ in the first epoch followed by *f*_2_ in the second epoch; Target 4 is coded only by presenting *f*_2_ in the two epochs. Figure 4 indicates that the targets can be coded either by a specific single frequency (Target 1 by only *f*_1_ and Target 4 by *f*_2_), or by the different presentation of two frequencies(Target 2 by *f*_1_ -*f*_2_ and Target 3 by *f*_2_-*f*_1_). Obviously, if bits 0 and 1 are used to denote *f*_1_ and *f*_2_ respectively, the temporal coding sequence just like the binary code technique in information science. For Figure 4, the four targets are corresponding to 0 0, 0 1, 1 0 and 1 1, respectively. The break time epoch is used to provide the time for subject to shift gaze.

### 3 Stimulator

**Figure 5:**
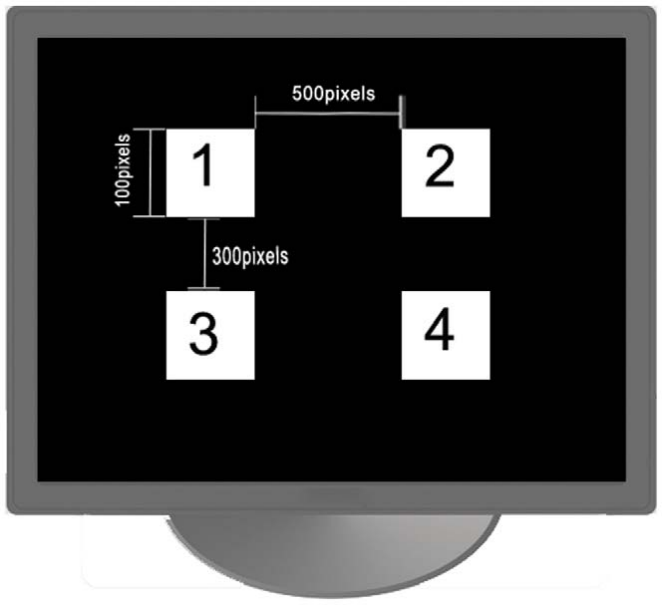
The distribution of four targets on the computer screen.

In this study, a Lenovo 19″ LCD screen with the resolution of 1280H×768 V Pixels, and a refresh rate of 60 Hz was used to present stimuli. Four flickering targets were displayed at four positions of a LCD monitor. The stimulus flickers were controlled by a computer through a control program written in C++ builder based on Windows DirectX API. Two frequencies, 7.5 Hz and 12 Hz, that can evoke strong SSVEPs, are chosen to code the four stimuli under MFSC paradigm. Each stimulus was presented in a 100×100 pixels square and the space between stimuli was 500 pixels in horizontal direction and 300 pixels in vertical direction. Figure 5 shows the distribution of the four targets on the computer screen. The flickering of stimuli was synchronized with the screen refreshing.

To accomplish the MSFC coding, the stimulator needs to send a trigger tag at the onset of each time epoch (*t*_1_, *t*_2_, *t*_3_) in one coding cycle to the EEG amplifier as an independent trigger channel, which can be used to segment the epochs for further analysis. A Symtop Amplifier (Symtop Instrument, Beijing, China), which has a parallel port for trigger synchronization, was used in our system. The three trigger tags for the time epoch (*t*_1_, *t*_2_, *t*_3_) were marked as 1, 2 and 3, respectively.

### 4 Experiment and Setups

The experiment was carried out in a normal room without electromagnetic shielding. The subjects were seated in a comfortable armchair, 60 cm away from the centre of the LCD monitor. An elastic cap with 16 Ag/AgCl electrodes arranged according to the international 10–20 system was used for EEG recordings with Symtop Amplifier (Symtop Instrument, Beijing, China), linked-earlobes are adopted as reference [34]. The parameters for the collection were: hardware filter between 0.5 and 45 Hz, sampling frequency 1000 Hz, and a 50 Hz notch filter for the line frequency interference (50 Hz in China), and impedance kept below 5 kΩ. The EEG signals were read out, stored and further processed with the self-developed software programmed in C++ builder. The recording electrodes Fp1, Fp2, F7, F3, F4, F8, T3, C3, C4, T4, T5, P3, P4, T6, O1, O2 were evenly distributed on the head surface. P3, P4, O1 and O2 reported to usually have strong SSVEP waveforms [19], [23], [24] were used in current study for CCA based frequency recognition. All the raw EEG signals were recorded for further offline analyses.

Ten paid healthy male right-handed subjects participated in this study. All of them had normal or corrected to normal vision. The age of the ten subjects ranged from 23 years to 30 years, with a mean of 24.5 years. These subjects had no risk of epileptic seizure. Seven of them were naive to the SSVEP-based BCI equipment and paradigm.

In the experiment, we presented four stimuli (targets) coded with two frequencies (7.5 Hz and 12 Hz) following MFSC protocol, and equal epoch durations for *t*_1_ and *t*_2_ are used. Most of current studies reveal that for the frequency to be reliably discerned, the time duration for frequency recognition needs to be more than 1 second [17], [19], [24], [25]. Accordingly, we set two conditions, i.e., 1.5 seconds and 2 seconds, for the duration of *t*_1_ and *t*_2_ (*t*_1_ = *t*_2_) to carry out our experiment. In each condition, the experiment run two sessions with each session containing 10 trails for each of the four stimuli, resulting in 40 trials for each session. Based on the used duration time of 1.5 seconds and 2 seconds, each trail lasted for 3 seconds, or 4 seconds followed with a 3 s rest time (*t*_3_). In each session, the stimulus sequence of the four targets was random, but the presentation order of stimuli was consistent between different condition and subject. Subjects were asked to gaze at a predefined flickering stimulus during each trial, where the digital number of the target that the subject should gaze at during next trial was presented on the center of the LCD monitor in the rest period. Subjects have a 2–3 minutes break between each session to relax. The experiment lasted for about 1 hour.

### 5 Target recognition for MFSC

Based on the above introduction, when MFSC is used to code targets, the strategy used for target recognition is to detect which coding sequence is involved in the current task. To perform the coding sequence detection, we need to know the onsets (*t*_i_, *i* = 1,2,3) of one cycle, and it is a problem to automatically detect the onset in practice. In this work, we can easily resolve this problem by sending the trigger tags at the onset of each time epoch (*t*_1_, *t*_2_, *t*_3_) in one coding cycle to the EEG amplifier, which can embed the tags in recordings.

**Figure 6:**
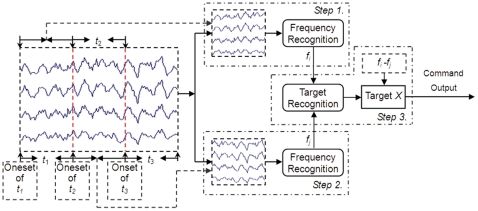
The target recognition diagram for EEG data in one cycle under MFSC protocol.

During our off-line analysis, all recordings were preprocessed as stated in the part of *Results*. Then, with the epoch tags (1, 2 and 3), the recordings were segmented into cycle trials, and epochs 1 and 2 are further determined by sub-dividing cycle trial according to tags 1–2 epoch *t*_1_, tags 2–3 for epoch *t*_2_, and tags 3-1 for epoch *t*_3_. The EEG at P3, P4, O1 and O2 are used for CCA analysis. The EEG during epoch *t*_3_ is excluded for further analysis. Based on the above preprocessing, the target gazed in each coding cycle can be recognized following the procedure in Figure 6.

To determine the coding sequence in one cycle, the frequency recognition algorithm needs to recognize the flickering frequency in each epoch. The procedure of frequency recognition in one cycle (Figure 6) can be summarized as:

Step 1. Recognition frequency in epoch *t*_1_. The EEG data in epoch *t*_1_ was input to the frequency recognition algorithm to determine the dominated frequency (*f*_i_) in current epoch;

Step 2. Recognition frequency in epoch *t*_2_. Similar to that in Step 1, the dominated frequency (*f*_j_) was estimated based on the EEG in epoch *t*_2_;

Step 3. Target recognition. After getting *f*_i_ and *f*_j_, we had the coding sequence *f*_i_-*f*_j_, then compared it with the coding sequences of all the targets. The target having the same coding sequence is regarded to be gazed by subject, and the corresponding command was sent to the application interface of the BCI system.

In Steps 1 and 2, the frequency recognition methods like FFT [4] and canonical correlation analysis (CCA) [23] could be used to detect the frequency. Because of the good performance of the primary CCA [19], [30], [31], it was adopted in this work.

CCA is a multivariable statistical method to seek linear combinations so that two sets of data have maximum correlation with each other. CCA extends ordinary correlation to two sets of variables. It had been used in neuroimage data analysis [23], [35].When using CCA for frequency recognition, we have to create several reference signals. There are two main parameters for the reference signals, data length and the number of harmonics. Data length of the reference signals is equal to the number of sampling point of EEG and the number of harmonics is dependent on how many frequency harmonics can be evoked by SSVEP stimulus.

Let *X* be the multiple channel signals of EEG, *Nt* be the number of sampling points, and *F_s_* be the sampling rate. When the stimulus frequencies used for SSVEP based BCI system are *f*_1_, *f*_2_,…, *f_L_*, we can use the frequency information to create the reference signals as
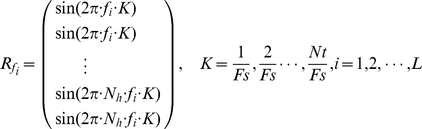
(1)where *N_h_* is the number of harmonics. The involvement of the harmonics in the reference signals is due to the fact that SSVEP is usually observed with the same fundamental frequency of the driving stimulus as well as its harmonics [1]. In this work, the number of harmonics is 2.

With CCA, we can find the weight vectors *W_x_* and *W_r_* to get the maximum canonical correlation of *X* and *R_fi_* (*i* = 1,2,…*L*) by solving the following optimization problem
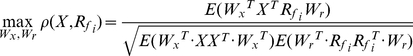
(2)For each reference signal, we can get a maximum canonical correlation, then used these coefficients to recognize the target which the user attended in the SSVEP-based BCI system as
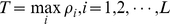
(3)Where 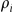 are the CCA coefficients obtained with the *L* reference signals.

Although we can get the second largest coefficient, third largest coefficient, et al., only the maximum correlation coefficient was used as the classification basis in present work as proposed in [23]. CCA can be implemented by the *M* function of Matlab (Mathwork) canoncorr.m. For the details about CCA, please refer to [23], [28], [35].

### 6 Evaluation index

The main evaluating index for the BCI system is the bit rate (the amount of information communicated per unit time) which is the standard method for measuring communication and control systems [20]. Bit rate depends on both speed and accuracy. The accuracy in BCI system is the ratio of the correct recognition commands divided by all the commands desired to send by the users with the system. If a trial has *N* possible targets in SSVEP-BCI system with each target having the same probability of being desired by the users, if the probability *P* that the desired target will actually be selected is always the same, and if each of the other (i.e., undesired) targets has the same probability of being selected (i.e. (1−*P*)/(*N*−1)), the bit rate (in bits/minute) can be computed as follows:
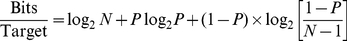
(4)

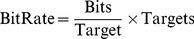
(5)


### Conclusion

This study proposed a new coding protocol for SSVEP based BCI, which uses the temporal order information of sequential flickers to code targets. The test in this study confirmed its effectiveness as an adequate application of the limited available frequencies to code more targets for the SSVEP-based BCI. Future work needs to construct a speller system with high ITR under MFSC protocol.

## References

[1] Regan D (1989). Human brain electrophysiology: evoked potentials and evoked magnetic fields in science and medicine:.

[2] Middendorf M, McMillan G, Calhoun G, Jones KS (2000). Brain-computer interfaces based on the steady-state visual-evoked response.. IEEE Trans Rehabil Eng.

[3] Wang Y, Gao X, Hong B, Jia C, Gao S (2008). Brain-computer interfaces based on visual evoked potentials.. Engineering in Medicine and Biology Magazine, IEEE.

[4] Cheng M, Gao X, Gao S, Xu D (2002). Design and implementation of a brain-computer interface with high transfer rates.. Biomedical Engineering, IEEE Transactions on.

[5] Wu Z, Lai Y, Xia Y, Wu D, Yao D (2008). Stimulator selection in SSVEP-based BCI.. Med Eng Phys.

[6] Vialatte FB, Maurice M, Dauwels J, Cichocki A (2010). Steady-state visually evoked potentials: focus on essential paradigms and future perspectives.. Progress in neurobiology.

[7] Pfurtscheller G, Solis-Escalante T, Ortner R, Linortner P, Muller-Putz G (2010). Self-Paced Operation of an SSVEP-Based Orthosis With and Without an Imagery-Based “Brain Switch:” A Feasibility Study Towards a Hybrid BCI.. Neural Systems and Rehabilitation Engineering, IEEE Transactions on.

[8] Xu P, Yang P, Lei X, Yao D (2011). An Enhanced Probabilistic LDA for Multi-Class Brain Computer Interface.. PLoS One.

[9] Donchin E, Spencer KM, Wijesinghe R (2000). The mental prosthesis: assessing the speed of a P300-based brain-computer interface.. Rehabilitation Engineering, IEEE Transactions on.

[10] Panicker R, Puthusserypady S, Sun Y (2011). An Asynchronous P300 BCI with SSVEP-Based Control State Detection.. IEEE Trans Biomed Eng.

[11] Herrmann CS (2001). Human EEG responses to 1–100 Hz flicker: resonance phenomena in visual cortex and their potential correlation to cognitive phenomena.. Experimental brain research.

[12] Srihari Mukesh TM, Jaganathan V, Reddy MR (2006). A novel multiple frequency stimulation method for steady state VEP based brain computer interfaces.. Physiol Meas.

[13] Shyu KK, Lee PL, Liu YJ, Sie JJ (2010). Dual-frequency steady-state visual evoked potential for brain computer interface.. Neurosci Lett.

[14] Lee PL, Sie JJ, Liu YJ, Wu CH, Lee MH (2010). An SSVEP-actuated brain computer interface using phase-tagged flickering sequences: a cursor system.. Ann Biomed Eng.

[15] Cecotti H (2010). Spelling with Brain-Computer Interfaces-Current trends and prospects.. Proceedings of 5th French Conference on Computational Neuroscience.

[16] Jia C, Gao X, Hong B, Gao S (2011). Frequency and phase mixed coding in SSVEP-based brain–computer interface.. IEEE Trans Biomed Eng.

[17] Cecotti H (2010). A self-paced and calibration-less SSVEP-based brain-computer interface speller.. IEEE Trans Neural Syst Rehabil Eng.

[18] Yan Z, Gao X (2011). Functional connectivity analysis of steady-state visual evoked potentials.. Neuroscience letters.

[19] Bin G, Gao X, Yan Z, Hong B, Gao S (2009). An online multi-channel SSVEP-based brain-computer interface using a canonical correlation analysis method.. J Neural Eng.

[20] Wolpaw JR, Birbaumer N, McFarland DJ, Pfurtscheller G, Vaughan TM (2002). Brain-computer interfaces for communication and control.. Clin Neurophysiol.

[21] Teng F, Choong AM, Gustafson S, Waddell D, Lawhead P (2010). Steady state visual evoked potentials by dual sine waves.. Proceedings of the 48th ACM Southeast Conference.

[22] Friman O, Volosyak I, Graser A (2007). Multiple channel detection of steady-state visual evoked potentials for brain-computer interfaces.. IEEE Trans Biomed Eng.

[23] Lin Z, Zhang C, Wu W, Gao X (2006). Frequency recognition based on canonical correlation analysis for SSVEP-based BCIs.. IEEE Trans Biomed Eng.

[24] Wang Y, Wang R, Gao X, Hong B, Gao S (2006). A practical VEP-based brain-computer interface.. Neural Systems and Rehabilitation Engineering, IEEE Transactions on.

[25] Wu Z, Yao D (2008). Frequency detection with stability coefficient for steady-state visual evoked potential (SSVEP)-based BCIs.. J Neural Eng.

[26] Hardoon DR, Shawe-Taylor J (2008). Sparse canonical correlation analysis.. Machine Learning.

[27] Melzer T, Reiter M, Bischof H (2003). Appearance models based on kernel canonical correlation analysis.. Pattern Recognition.

[28] Brillinger DR (2001).

[29] Hastie T, Tibshirani R, Friedman J, Franklin J (2009). The elements of statistical learning: data mining, inference and prediction..

[30] Bin G, Gao X, Wang Y, Li Y, Hong B (2011). A high-speed BCI based on code modulation VEP.. Journal of neural engineering.

[31] Wang YT, Wang Y, Jung TP (2011). A cell-phone-based brain-computer interface for communication in daily life.. Journal of neural engineering.

[32] Ding J, Sperling G, Srinivasan R (2006). Attentional modulation of SSVEP power depends on the network tagged by the flicker frequency.. Cereb Cortex.

[33] Pfurtscheller G, Allison BZ, Brunner C, Bauernfeind G, Solis-Escalante T (2010). The hybrid BCI.. Frontiers in neuroscience.

[34] Yao D (2001). A method to standardize a reference of scalp EEG recordings to a point at infinity.. Physiological Measurement.

[35] Friman O, Cedefamn J, Lundberg P, Borga M, Knutsson H (2001). Detection of neural activity in functional MRI using canonical correlation analysis.. Magnetic Resonance in Medicine.

